# Management of cultural tourism in Ban Toon, Muang District, Phayao Province, Thailand during the COVID-19 pandemic

**DOI:** 10.12688/f1000research.134517.1

**Published:** 2024-02-09

**Authors:** Warach Madhyamapurush, Krantharat Khawatkun, Phatpitta Sreesoompong, Nattawut Somyarone, Wongduan Sakboonruang

**Affiliations:** 1 Department of Tourism and Hotel Management, University of Phayao, Mueang Phayao District, Phayao, Thailand; 2 Department of Communication Management, University of Phayao, Phayao, Thailand; 3 School of Business Management and Communication Arts, University of Phayao, Mueang Phayao District, Phayao, Thailand

**Keywords:** Tourism management, cultural tourism, COVID-19 pandemic, Ban Toon, Muang District, Phayao Province

## Abstract

**Background Add ABSTRACT above the background:**

The research aims to study the process of managing cultural tourism in the community and assess the socio-economic impact on the community with the help of eight key contributors to the project who play an essential role in the implementation of cultural tourism management.

**Methods:**

We prepared field notes from interviews and observations, and provided details from data collection (in the form of transcripts). Thematic analysis was employed to answer the research questions using a content analysis tool to obtain the community context in cultural tourism and tourism management dimensions. Participatory Action Research (PAR) was employed to meet the needs of stakeholders and test tourism routes through such routes. The tourism route development was evaluated using descriptive statistics.

**Results:**

There is one new cultural tourism route and the process of managing cultural tourism developed community-level organizations with spiritual leaders as the main advisors. The result of the net present value (NPV) analysis is 917,149 baht or 27,792$. The return from the implementation of this project (the internal rate of return (IRR)) is 11.32%. In addition, the ratio of the sum of the present value over the life of the project to the sum of the present value of the cost over the life of the project is 2.45 times, giving the project, if continued, an income 2.45 times greater than its expenditure.

**Conclusions:**

Cultural tourism management in Ban Toon, Muang District, and Phayao Province during the COVID-19 pandemic can be used as a guideline in other communities with similar contexts.

## Introduction

The COVID-19 pandemic from 2020–2022 caused many changes, especially regarding travel. The tourism industry was greatly affected, particularly the main tourist activity of traveling and socializing. The local government should assist the business sector in helping communities develop sustainably in an Albanian location where tourism has been affected by COVID-19 ( Alshiqi & Sahiti, 2022). A Spanish case study ( Rodríguez-Vázquez *et al.*, 2023) demonstrates that cultural tourism is a more potent draw than other types of tourism. To comprehend the potential of cultural tourism to help resolve the issue and its reaction to the needs of new post-pandemic visitor behavior, it may still be essential to pinpoint the critical factors. The business structure of the tourism sector may need to be modified in light of the current circumstances to handle the crisis, protect the company, and protect jobs. This indicates that a thorough plan is needed to resuscitate the tourism sector. Future research should examine the effects of the COVID-19 outbreak on incoming and outward travel, visitor behavior, employment, and the industry’s overall growth rate ( Santus Kumar DEB, Shohel Md. NAFI, 2020).

The Thailand Safety and Health Administration (TSHA) has released measures in collaboration with the Tourism Authority of Thailand, the Ministry of Tourism and Sports, and the Department of Disease Control in the Ministry of Public Health to protect and foster confidence in public health safety. These initiatives increase visitor comfort levels and make tourist destinations safer ( Rodríguez-Vázquez *et al.*, 2023). The COVID-19 epidemic affected many of Thailand’s community tourist attractions, even though many were approved to meet specific standards, according to a study ( Rodríguez-Vázquez *et al.*, 2023). These closures resulted in a loss of revenue for the neighborhood. As a result, those who supplied tourist-related services either changed the type of work they were doing or moved to work somewhere else (or both), leaving previously dependent regions on tourism with few other options for income ( Kofi Poku Quan-Baffour, 2020).

Tourism is essential to the development of Thailand, especially its economic and social aspects. In economics, tourism generates income and promotes the distribution of funds based on the number of tourists to local areas. This, in turn, enhances job creation and employment in various occupations, directly and indirectly. Societally, tourism contributes to the development of public utilities and various facilities that help enhance the quality of life throughout the community. Tourism also helps communities and localities effectively revitalize and conserve natural and cultural resources. Furthermore, tourism also brings in experienced tourists, who help improve the quality of life and society.

One of the regions impacted by the decline in tourism brought on by the COVID-19 outbreak is Phayao Province. The province announced an indefinite suspension of tourism activities, including a ban on travelers entering Phayao Province, as well as other measures to address the crisis that arose in the early stages of the pandemic. At the end of 2021, the government announced relief measures that allowed tourism operations to resume, but only with the SHA+ standard.

Because of its 2010 designation as a national model village with a self-sufficient economy, the Ban Toon community in Muang District, Phayao Province, has high tourism potential. The western area of the community is a source of agricultural learning, producing organic rice and being one of the suppliers of pickled fish in Phayao Province. This has increased the number of tourists interested in and learning about agricultural methods. In addition, the researchers’ spatial survey results showed that the eastern portion of the province had a natural, historical tourist attraction. The ancient port, with cruise activities to see the Red Lotus Sea, is considered one of the most crucial community tourist attractions in Phayao Province. In the two and a half years from 2017 to 2020, there were approximately 3,500 tourists, consisting of study groups visiting Phayao Lake 100 times per year, with roughly ten people per group, totaling 1,000 people per year who came to see the Red Lotus Sea in Phayao Lake. The estimated annual income was as follows: The income from providing educational visits costs 200 baht per person (6.06$), or 200,000 baht per year (6,061$). General tourist groups had expenses related to accommodation (homestay), food, and souvenirs (organic rice), averaging 500 baht per person (15.15$), totaling 1,000,000 baht per year (30,303$). While additional activities specifically to see the Red Lotus Sea earned an average of 100 baht per person (3$), totaling approximately 1,250,000 baht (37,878.79$). However, a survey of tourism conditions in early 2021 found that tourism, which had previously generated an income of approximately 1,250,000 baht per year (37,878.79$), had decreased to only 100,000 baht (30,303$). The number of tourists who had previously visited Phayao Province was estimated to be 3,500 per year. It was found that this number decreased to a mere 200 people per year during the pandemic. As a result, people in the community ceased to operate tourism activities, thus further reducing the likelihood that tourists would visit Phayao Province and the Red Lotus Sea area.

Phayao Province planned a four-year (2017–2021) “Phayao Wellness” initiative. A part of the government’s mandated COVID-19 relief measures was the SHA+ standard for regulating tourism activities. Thus, the question became, during the pandemic, how would the Ban Toon community in Phayao manage cultural tourism so that people could earn a living? The project “Cultural Tourism Management, Ban Toon, Muang District, Phayao Province, during the COVID-19 Pandemic” set out to answer this question. This study aimed to evaluate and develop the process of cultural and tourism management. It includes creating cultural tourism routes and designing cultural products that would facilitate the cooperation of Ban Toon villagers throughout the sub-district. Additionally, the economic and social impacts of the project were assessed.

### Research objectives

1. To study the context and readiness of the Ban Toon Sub-District community area.
2. To develop cultural tourism routes and cultural tourism management.
3. To assess the economic and social impacts of cultural tourism management.

### Literature reviews

The concept of tourism systems can be understood by studying related concepts, such as Dickman’s (1996) and Leiper’s (1990) travel composition concepts on tourism systems. Tourism management must consider the host dimension, whereas the source mainly considers the needs of tourists. Madhyamapurush’s (2019) work on the tourism system studied the entire tourism project across three dimensions: the demand side, the response or supply side, and the support side. Madhyamapurush’s study includes the work of Goeldner and Ritchie (2006), defining the components of tourism, or the “tourism phenomenon,” of tourism and tourism management. This includes Kušen’s (2010) work on the tourism system of tourist attractions. A tourism attraction system developed by studying all five researchers attempted to explain tourism links in a global context. Dickman (1996) and Kušen (2010) mainly focused on how attractions should have essential components. Leiper (1990) and Madhyamapurush (2019) linked a knowledge set of tourist needs to create responses to those needs in tourism activities. In addition, Goeldner and Ritchie (2006) and Madhyamapurush (2019) provided an appropriate administrative process or method for demand, supply, and support. These provide a broader picture of the tourism structure and have not yet been effectively applied to community tourism. The study also found that a private agency primarily provides the response side (supply side).

The concept of tourism management has been developed from a business system to enable the tourism activities of government agencies, the private sector, and community tourism to be able to conduct activities appropriately, using the original management concept of Fayol (1916): POCCC (Planning, Organizing, Commanding, Coordinating, and Controlling). Moreover, it relies on the concept of co-management, the system tourism ( Madhyamapurush, 2018) described for the tourism management model used in Phu Hin Rong Kla National Park, Phitsanulok Province, which proposed the idea of cooperation between the government and the tourism community in the national park to direct the work there.

Tourists gain experience in tourism through the service provided by the provider and the experience that comes from learning about, getting in touch with, or participating in activities in tourist areas. The study of tourism experience factors was divided into two parts. The first designers to study experience management utilized the concept of Pine and Gilmore (1998). This consisted of four areas of relationship - entertainment, education, aesthetics, and escape – along with two dimensions of participation: participation and relationship with the environment. These concepts were combined with those of Gao, Scott, & Ding (2010) and Cetin & Bilgihan (2015), which consist of outline, atmosphere, memorable story, interaction, service, culture, heritage, and challenges. For the tourist experience, the psychological concepts of Poulsson and Kale (2004) were used, which consisted of novelty, learning, personal relationships, surprise, and participation. The study of the relationship between these two components makes up for Cutler and Carmichael (2010).

The economic theory (Growth Model) of Harrod-Domar ( Besomi, 2001) ( Mahawong, 2001) has developed a theory or model of economic and social impact assessment. Economic growth aims to describe the relationship between the two within a particular level of growth. Both concepts hold that investments are at the heart of economic growth. In addition to increasing revenue, there is also the ability to increase efficiency. The production of the economy and maintaining full employment require economic growth that is high enough to absorb savings, which is desired for income investing at the whole employment level. Harrod-Domar assumes that the model is either an industrial unit of production or that the economy as a whole depends on the amount of capital invested in that economic unit.

### Conceptual framework

**Figure 1.**
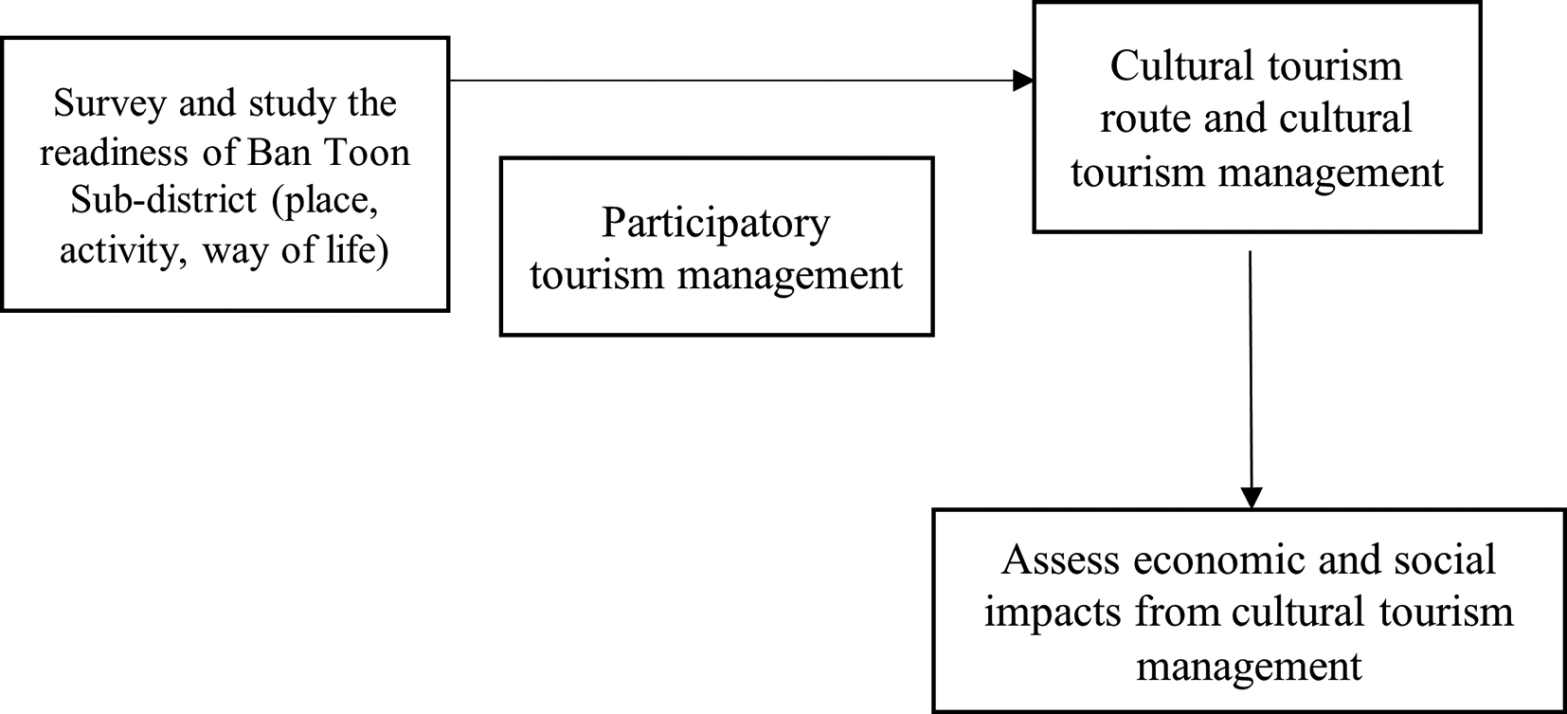
Demonstrates the Conceptual framework of this research. Source: Researcher's synthesis.

## Research methods

### Ethics

The Human Ethics Committee of the University of Phayao approved the research protocol (approval number UP-HEC 2.2/078/64); the date of approval was August 22, 2021. Participants were given the purpose of the research and asked to participate in the study; they welcomed it. Written consent was obtained from all participants, and they were informed that their anonymized data would be published. All participants also gave their permission and received information about the article’s methodology.

*Study design*

The research was carried out between August 20 ^th^ and 21 ^st^, 2021. General survey data on tourism resources were collected on September 21 ^st^. Interviews with stakeholders were conducted at Ban Toon on October 21 ^st^. Training on the tourism route and experience was conducted in Ban Toon on November 21 ^st^. The selection of tourist volunteers was completed on December 21 ^st^. Testing of tourism routes with tourist volunteers and data collection was conducted on January 20 ^th^ and 22 ^nd^. Data were collected to assess the economic and social impact of stakeholders in the community on March 22 ^nd^.

*Sampling*

Key informants included local sages, community leaders, community product operators, attraction supervisors, and community development officers of the Ban Toon Sub-District Administrative Organization. They were selected using purposive sampling to obtain accurate cultural information. The selection of volunteer tourists or simulated tourists included announcements for interested applicants to apply via Facebook. The qualifications of volunteers are that they must not be a person in the study area and must have an understanding of the evaluation.

The study utilized an advertisement posted on Facebook to attract tourists’ interests and encourage their participation. The advertisement was shared by researchers’ colleagues and other associates, expanding its reach and visibility among potential participants. As tourists came across the advertisement on Facebook, they found it interesting and decided to participate in the study on the management of cultural tourism in Ban Toon. By leveraging the power of social media and utilizing networks within their professional circles, researchers were able to effectively promote their studies and generate interest among potential participants. Many tourists discovered the advertisement and found it intriguing. Consequently, they decided to participate in the study.

The researchers used Facebook as a platform to make announcements because of its widespread reach and the potential to attract a diverse pool of applicants. By utilizing Facebook, we aimed to engage individuals who were interested in participating as volunteers or simulated tourists in the study. To select participants, the researchers employed a method in which they considered the acceptance requests received through Facebook as the basis for their selection process. Individuals who expressed their interest and were formally requested to participate via Facebook were chosen as study participants. As a result, the study’s participant pool was limited to individuals who had submitted an acceptance request through Facebook. The use of Facebook as the primary channel for recruitment and participant selection plays a significant role in determining the composition of the study sample. It is important to note that using Facebook as a recruitment platform may introduce certain limitations such as potential selection bias and limited representation from individuals who may not have access to or actively use the platform. Researchers have considered these factors when interpreting the findings and generalizing the results of the study.

*Data collection*

Questionnaire: A questionnaire was designed to gather data on the socio-economic impact of cultural tourism on the community. It was distributed to community members, including tourists, and community leaders ( Madhyamapurush, 2023).

Interviews: In-depth interviews were conducted with ten key contributors to the cultural tourism management project, including spiritual leaders, community leaders, and tourism business owners. These interviews provided valuable insights into the process of managing cultural tourism in the community ( Madhyamapurush, 2023). The interviews were conducted by the research team, which consisted of several individuals with expertise in cultural tourism and socio-economic impact assessment. The interviews were conducted in person at the Ban Toon district. The interviews were semi-structured and followed the COREQ guidelines to ensure that all necessary information was gathered. The interviews were audio-recorded with the participants’ consent, and detailed field notes were taken during and immediately after each interview.

Route Testing: Participatory Action Research (PAR) was used to test the tourism routes in the community. This approach allowed stakeholders to be involved in the development and testing of the routes. The tourism route development was evaluated using descriptive statistics.

For the data collection process, the researchers involved ten key contributors to the project who played an essential role in the implementation of cultural tourism management in Ban Toon, Muang District, Phayao Province, Thailand. Key informants, such as local sages, community leaders, community product operators, attraction supervisors, and community development officers from the Ban Toon Sub-District Administrative Organization, were selected using a purposive sample to gather accurate cultural data. A total of ten people were chosen. Additionally, 30 tourists tested the route, with applications accepted for those traveling in November 2021. The data collection process involved several stages. Firstly, the researchers conducted interviews with the key contributors to gather their perspectives and insights on the process of managing cultural tourism in the community during the pandemic. The interviews were conducted in-person and via video conferencing tools such as Zoom. Secondly, the researchers conducted observations of the community to gain a better understanding of the community’s context in cultural tourism and tourism management dimensions. The observations were conducted on-site and involved the researchers actively participating in the community’s cultural tourism activities. Thirdly, the researchers prepared field notes from the interviews and observations that were conducted. The field notes were used to provide details for the data collection process and served as a reference during the analysis stage. Lastly, the data collection process involved the use of Participatory Action Research (PAR) to meet the needs of stakeholders and test tourism routes through such routes. The tourism route development was evaluated using descriptive statistics. Overall, the data collection process involved a collaborative approach between the researchers and the key contributors to the project. The researchers worked closely with the contributors to ensure that the data collection process was comprehensive and accurate.

The research utilized Participatory Action Research (PAR) to develop a cultural tourism model that meets the needs of tourists and to test the tourism routes. The tourism route assessment form ( Madhyamapurush, 2023) was created. The validity of the questionnaire was examined carefully. Three experts examined each of the questionnaire items. The content validity and linguistic suitability were evaluated using an Index of Item Objective Congruence (IOC). An IOC score of 0.70 indicates the permissible range. The questionnaire was then altered in accordance with the experts’ recommendations, and reliability was evaluated using Cronbach’s alpha coefficient. The result of 0.948 was deemed statistically significant.

The researcher prepared field notes from interviews and observations and provided details from data collection (in the form of transcripts) ( Madhyamapurush, 2023). When volunteers arrived, the researcher explained the tourism process. Villagers conduct tourism along the developed tourism route. Researchers created assessment forms for volunteers. The volunteers in this study conducted the assessment of the participants. Researchers conducted interviews on the use of travel routes.

*Data analysis*

Methods for processing data prior to and during analysis:

- Transcription: The interviews were transcribed verbatim, including all pauses, hesitations, and nonverbal cues.
- Data entry: The transcripts were entered into a computer and stored in a secure database.
- Data management and security: The database was password-protected and only accessible to authorized personnel. The data was backed up regularly to prevent loss or corruption.
- Verification of data integrity: The transcripts were cross-checked for accuracy and completeness by two independent researchers.
- Data coding: The transcripts were coded using a content analysis tool to identify themes and patterns related to the research questions.
- Anonymization/deidentification of excerpts: Any identifying information, such as names or locations, was removed from the transcripts to protect the privacy of the participants.
- The researchers involved in data analysis were trained in qualitative research methods and had experience in analyzing data from similar studies.
- The rationale for using this approach was to allow for a more in-depth understanding of the experiences and perspectives of the participants.

Descriptive statistics were used to analyze the collected data, which was done using SPSS Version 27.0. To address the research questions and gain insight into the cultural tourism and tourism management dimensions within the community, a thematic analysis method was employed through a content analysis tool. Content analysis was used to create cultural tourism routes, allowing tourists to travel independently and engage with the Ban Toon community. The development of these tourism routes was evaluated using descriptive statistics.

## Results

### Context and readiness of the area

The study found that “Ban Toon is a community that has cultural road activities, consisting of routes that concretely reflect the distinctive local cultural identity, such as old houses, a market that still maintains a local way of life, temples, ancient sites, religious places, and various artistic activities, as well as local wisdom” ( Rodríguez-Vázquez *et al.*, 2023). In addition, the Ban Toon community has its roots in the Tai Yuan ethnic group, a population group that settled in northern Thailand (Chiang Saen) and migrated to the area of the Ban Toon Sub-District until it became a large community with a strong local culture. Various customs are considered a part of the intangible cultural heritage of the Ban Toon community. These consist of the following:

1. Food culture. Ban Toon is an urban community that continues to cook food using traditional methods and culture. The community has outstanding local dishes, such as northern spicy curry and fish curry.
2. Clothing culture. The general style of dress of Ban Toon village men is three-quarter length pants (troops) made of cotton-dyed blue or black. Shirts are round-necked, short-sleeved cotton with small slits on either side (the Mahom shirt). Women dress in a sarong that almost reaches their ankles. The edge of the sarong has a beautiful pattern. The shirt was round-necked with colorful and beautiful patterns.
3. Housing culture. Buildings in the Ban Toon community maintain traditional Lanna (northern style) architecture. Many new residential houses are still in the original style of ancient houses.
4. Traditional culture. The Ban Toon community continues to maintain various traditions, such as setting up the Thamma Luang and the Mahachat Sermon (Vessantara Jataka), held during the festival of floating lanterns in the Yi Peng month or the 12th lunar month.
5. Language culture. The villagers of Ban Toon still use the Northern Thai dialect (Kham Muang language) and the Lanna script. The ancient scriptures of the Ban Toon Tai Moral Community are preserved and sorted into categories.
6. Occupation culture. Most baboon villagers are engaged in agriculture. Local handicrafts also include wickerwork, products made from water hyacinths, lanterns, and ancient flags.
7. Belief culture. The Ban-Toon community is an ethnic community that has migrated from Chiang Saen. Therefore, various beliefs are based on the Lanna culture, such as ancestral spirit raising and traditions to support the buffalo. In addition, there are also important Lanna rituals, namely, the ceremony to forgive or release misfortune, known as Su Kwan, which uses equipment that the villagers call “Satuang” when performing the ritual.
8. Performing art culture. The Ban Toon community has established a cultural learning center to transfer and train youth in the community to preserve Lanna’s traditional Thai music (Salor Saw Sueng).

The characteristics of the Ban Toon community reflect the agricultural nature of the area, which is connected to Phayao Lake and the Doi Luang National Park. Before the COVID-19 pandemic, visitors preferred agro-tourism activities in the western sub-districts and organic fields near the Doi Luang National Park entrance.

### Development of cultural tourism routes

**Figure 2.**
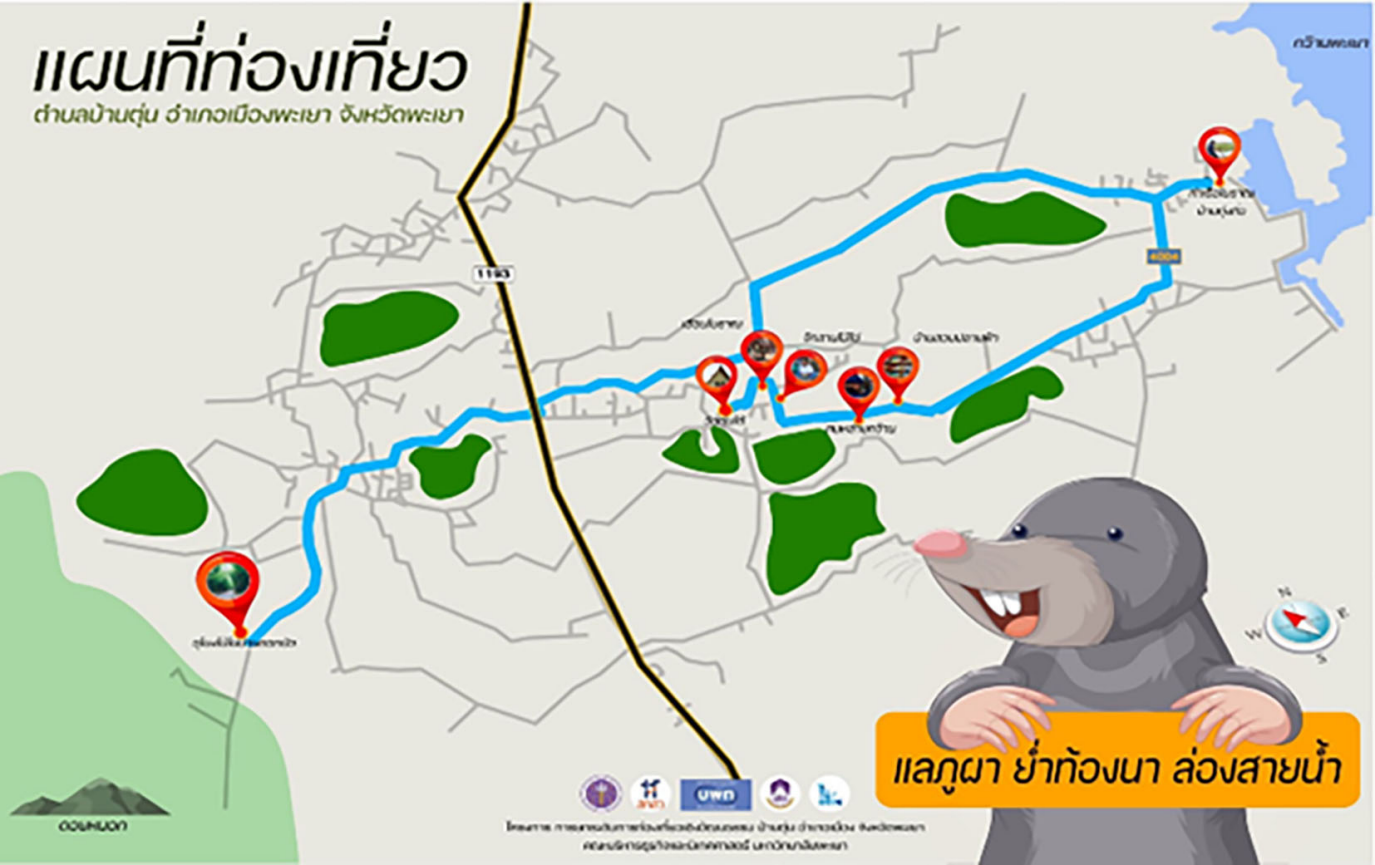
Shows the Tourist map “See the mountain, Walk through the rice fields, Cruise the river”. Source: Developed by a group of researchers together with the community.

Routes for cultural tourism are under development. The research team brought information to the initial meetings between community leaders and local communities. Information regarding the context of the area emphasized community participation in developing cultural tourism routes linking tourist sites. Tourism across the district is now more cohesive than it had been in the past. As a result, the tourism route connects the mountains, rice fields, and Phayao Lake based on the promotion slogan: “See the mountain, Walk through the rice fields, Cruise the river” ( Dickman, 1996). The cultural attractions of the Ban Toon community consist of Doi Luang National Park, Doi Nok, Huai Toon Reservoir, Phakretnark Waterfall, Ban Bua Bamboo Tunnel, Phu Klong Vineyard, Ban Toon Tai Temple, Ban Bua Temple, Analayo Temple, the clay pot learning center, So Huay House, the Ancient House, Ban Thung Kiew Ancient Ferry, and the local fishing group. The research team brought 30 tourists to the experimental group to test this route. Tourists gave their opinions, as shown in Tables 1 and 2, Figures 1 and 2.

**Table 1.** Shows the general information of tourists who tested the route.

General information	Number	Percentage
**Gender**		
Men	18	60
Women	12	40
**Age**		
15–20 years	8	27
21–25 years	5	16
26–30 years	9	30
31–35 years	5	16
36–40 years	3	10
**Number of hours touring the area**		
1–3 hours	12	40
4–6 hours	18	60

**Table 2.** Demonstrates the satisfaction evaluation of tourists towards the route - “See the mountain, Walk through the rice fields, Cruise the river”.

Issues in Education	Average	Meaning
Ease of access to tourist attractions (road signposts)	3.93	Excellent
Facilities in tourist attractions (restaurants, coffee shops, homestay accommodation (if desired), toilets, cell phone reception)	3.77	Excellent
Value and attractiveness of tourist attractions (tourist activities, beauty, community)	4.13	Good
Attractiveness of the environment (pollution levels, rich ecosystem)	4.33	Excellent
Management of tourist attractions (clarity of signposting and the starting point of the tourism route.)	4.23	Excellent
**Average**	4.08	Good

Tables 1 and 2 demonstrate that this tour route can take either 1–3 hours or six hours, indicating that the route can be adjusted to suit tourists’ travel time. The route is only suitable for a single day of travel. Moreover, the route had easy access to tourist attractions, with road routes and signposts averaging a satisfaction rating of 3.93. Facilities in tourist attractions (restaurants, coffee shops, homestay accommodation if desired, toilets, and cell phone signals) averaged 3.77. In contrast, the value and attractiveness of the tourist attractions (tourist activities, beauty, community) averaged 4.13. The environment of the tourist attractions (no pollution, complete ecosystem) averaged 4.33, and tourist attraction management (clarity of signposting and the starting point of the tourism route) averaged 4.23. The overall average of 4.08 implies that the tourists who tested the route had a high level of satisfaction, leading to the conclusion that the cultural tourism route “See the mountain, Walk through the rice fields, Cruise the river” is popular both with people in the community who developed the route and tourists who have come to experience it.

Through examination of the study of cultural tourism routes, the researchers found that fortune-telling activity is an essential ritual in the community and is commonly performed to increase merit and release bad luck. Understanding the cultural importance of such a ritual, the research team designed an activity in which participants, together with community leaders and others, assembled a merit-making set (Satuang), a cultural product of the Ban Toon community. This encourages tourists who come to experience the culture of the Ban-Toon community along the specified route. The Boon Teung set (Satuang) is a product created by people in the community to sell to people who live or work abroad and, in the coming years, will not be able to attend the ceremony, yet still wish to make merit and release bad luck.

Preparing the tourism route “See the mountain, Walk through the rice fields, Cruise the river” and activities for the Boon Teung (Satuang) event needed to be developed through a committee of a cultural tourism group in the Ban Toon community. The committee includes a mentor and a spiritual leader and chairs the official committee. It includes eight directors from local government agencies, other spiritual leaders, travel leaders, and community leaders who collaborate with tourism and community leaders through participatory work processes.

### Economic and social impact assessment of the cultural tourism route

An analysis ( Table 3) of the assessment of the economic and social impact of the research found that the project will have a net present value (NPV) income result of 917,149 baht (27,792.39$) in its first five years of operation. The internal rate of return (IRR) from the implementation of this project is 11.32%, which is higher than the bank deposit rate, making the project a viable investment. Moreover, when considering the ratio of the present value of results to the sum of the present value of costs throughout the project’s life, indications are that this project will yield up to 2.45 baht (2.45$) for every 1 baht invested (1$). While the net income will be 2.45 times greater than the expenditure, the estimated income will still be less than that of 2017-2020 due to the overall reduction in tourism since the COVID-19 pandemic.

**Table 3.** Shows the Evaluation of economic and social impacts of tourism routes.

Year	2022	2023	2024	2025	2026	Total
1. Revenue	($)	($)	($)	($)	($)	($)
Income from the sale of souvenirs	54.54	1204.55	1445.45	1686.36	1927.27	7227.27
Income from tourism in the area	4545.45	4545.45	4545.45	4545.45	4545.45	22727.27
Income from restaurants and coffee shops in the area	2424.24	3030.30	3636.36	4242.42	4848.48	18181.82
Reduction in local health expenditures (time costs, medication costs, travel costs)	1515.15	1515.15	1515.15	1515.15	1515.15	7575.76
Total reward	9448.48	10295.45	11142.42	11989.39	12836.36	55712.12
2. Cost						
2.1 Operating expenses						
- Cost of building a tourist route	7272.73	0.00	0.00	0.00	0.00	7272.73
- Cost of souvenirs	606.06	757.58	909.09	1060.61	1212.12	4545.45
- Food and coffee costs	1454.55	1818.18	2181.82	2545.45	2909.09	10909.09
Total cost	9333.33	2575.76	3090.91	3606.06	4121.21	22727.27

## Conclusion and discussion

The research team reached the following conclusions by studying cultural tourism management in Ban Toon, Muang District, Phayao Province, during the COVID-19 pandemic.

The context and readiness of the Ban Toon area, Muang District, Phayao Province, is a community with cultural road activities that consist of routes that reflect and express distinctive local identities, such as old houses. These markets maintain a local way of life, temples, ancient sites, religious sites, traditional culture, local wisdom, and various artistic activities. Furthermore, there is an aesthetic corresponding to the concept of Izidora Marković’s (2017) research on tourism areas, which found that sustainability in tourism management should consider the area’s management to create attractiveness for tourists, including preserving the area’s sustainability. This is consistent with the research of Keeraphan, Boonkhum, and Sungraksa (2016). The authors demonstrated that many Boon Toon environments and tourism resources were ready to host visitors, promoting education and naming a community leader responsible for tourism.

The development of the cultural tourism route, “See the mountain, Walk through the rice fields, Cruise the river,” connects natural and cultural attractions, as well as tourist attractions across the entire district, consisting of Doi Luang National Park, Doi Nok, Huai Toon Reservoir, Phakretnark Waterfall, Ban Bua Bamboo Tunnel, Phu Klong Vineyard, Ban Toon Tai Temple, Ban Bua Temple, Analayo Temple, the clay pot learning center, So Huay House, Ancient House, Ban Thung Kiew Ancient Ferry, and a local fishing group. Tourism activities can be scheduled to suit tourists’ preferences, which corresponds to Pine and Gilmore’s (1998) concept of experience design, which states that the four boundaries of the relationship are entertainment, education, aesthetics, and escape, along with the two dimensions of participation, participation, and relationship with the environment. This allows travelers to engage with service providers and have experiences that facilitate hands-on learning while participating in activities in tourist areas.

The management of cultural tourism has been developed in the Ban Toon community cultural tourism group with the establishment of a formal committee consisting of consultants, a spiritual leader, a chairman, and eight committee members from local government agencies working with tourism and community leaders through a participatory work process. This corresponds with Piyaporn Taweekul’s (2001) research, which proposed that sustainable community tourism management relies on specific main components. It includes community needs, opening up to tourism, and participation in tourism management.

After analyzing the net present value (NPV), the economic and social impact assessment found that the project will have a net income of 917,149 baht (27,792.39$) over the next five years. The internal rate of return (IRR) analysis showed a rate of 11.32%, which is higher than the bank deposit rate. This makes the project worthy of investment. Additionally, when considering the ratio of the present value of the results throughout the project’s life to the sum of the present value of its costs, the project continues to earn 2.45 times more than expenses.

Cultural tourism management in Ban Toon, Muang District, and Phayao Province during the COVID-19 pandemic can be used as a guideline in other communities with similar contexts. Tourism must have the potential and availability of resources and include the participation of people in the community to achieve a distribution of income while thoroughly developing community areas.

## Data Availability

Figshare: ‘Management of Cultural Tourism in Ban Toon, Muang District, Phayao Province, Thailand during the COVID-19 pandemic, https://doi.org/10.6084/m9.figshare.22683232.v3 ( Madhyamapurush, 2023). This project contains the following underlying data:
- Underlying Data - 24th April 2023.xlsx This project contains the following extended data:
- Questionnaire - Assessment form Data are available under the terms of the Creative Commons Attribution 4.0 International license (CC-BY 4.0).

## References

[ref21] Alshiqi S Sahiti A : The Impact of COVID–19 on Albanian Tourism. *Emerg. Sci. J.* 2022;6:33–41. Special Issue “COVID-19: Emerging Research”, 2022. 10.28991/esj-2022-SPER-03

[ref24] Besomi D : Harrod’s Dynamics and the Theory of Growth: The Story of a Mistaken Attribution. *Cambridge J. Econ.* 2001;25(1):79–96. 10.1093/cje/25.1.79

[ref1] Cetin G Bilgihan A : Components of cultural tourists’ experiences in destinations. *Curr. Issue Tour.* 2015. Ahead of print. 10.1080/13683500.2014.994559

[ref2] Cutler S Carmichael B : The Dimensions of Customer Experience. Morgan M Lugosi P Ritchie B , editors. *The Tourism in Leisure Experience: Consumer and Managerial Perspectives.* Aspects of Tourism: Bristol;2010; pp.3–26.

[ref3] Deb SK Nafi SM : Impact of COVID-19 Pandemic on Tourism: Recovery Proposal for Future Tourism. *Geoj. Tour. Geosites.* August 13, 2020;33(4 Supplement):1486–1492. Year XIIII. 10.30892/gtg.334spl06-597 Reference Source

[ref4] Dickman CR : *Overview of the Impacts of Feral Cats on Australian Native Fauna.* Canberra: Australian Nature Conservation Agency;1996.

[ref23] Fayol H : *General and industrial management.* London: Sir Isaac Pitman and Son;1916.

[ref6] Gao L Scott N Ding P : Design of memorable cultural heritage attraction experiences for tourists. *Paper presented at the Proceedings of the International Scientific Conference: Culture in the Integrated World.* 2010.

[ref7] Goeldner CR Ritchie BJ : *Tourism: Principles, practices and philosophies.* New Jersey: John Wiley & Sons;2006.

[ref9] Izidora Marković V : *Sustainability Issues in Management of Tourism in Protected Areas: Case Study of Plitvice Lakes National Park.* London: Palgrave Macmillan;2017.

[ref10] Keeraphan L Boonkhum W Sungraksa N : *The Development of Activity Management Model Cultural Tourism of the Thai Phuan Community to Promote Creative Learning Nakhon Nayok Province.* Veridian E-Journal, Silpakorn University (Humanities, Social Sciences, and Arts);2016; pp.2160–2201. 1906–3431

[ref11] Kofi Poku Quan-Baffour: Cultural tourism and socio-economic regeneration of rural communities: the Apo festival of Bono Takyiman, Ghana. *African Identities.* 2020;21:134–149. 10.1080/14725843.2020.1856644

[ref12] Kušen E : A system of tourism attractions. *Tour. Rev.* 2010;58(4):409–424.

[ref13] Leiper N : Tourist attraction systems. *Ann. Tour. Res.* 1990;17(3):367–384. 10.1016/0160-7383(90)90004-B

[ref14] Madhyamapurush W : Tourism Management in Phu Hin Long Kran Nation Park, Phitsanulok. *Parichart Journal, Thaksin University.* 2018;31(3):131–144. Reference Source

[ref15] Madhyamapurush W : Structure of Tourism System. *J. Thai Hosp. Tour.* 2019;14(1):94–102. Reference Source

[ref16] Madhyamapurush W : Management of Cultural Tourism in Ban Toon, Muang District, Phayao Province, Thailand during the COVID-19 pandemic.Dataset. *figshare.* 2023. 10.6084/m9.figshare.22683232.v3 PMC11043658 38665333

[ref22] Mahawong S : *The development of economic knowledge.* Graduate school: Srinakharinwirot University;2001.

[ref17] Pine BJ II Gilmore JH : Welcome to the Experience Economy. *Harv. Bus. Rev.* 1998;76:97–105. 10181589

[ref19] Poulsson SHG Kale SH : *The experience economy and commercial experiences.* Boston: Harvard Business;2004.

[ref20] Rodríguez-Vázquez C Castellanos-García P Martínez-Fernández VA : Cultural Tourism in a Post-COVID-19 Scenario: The French Way of Saint James in Spain from the Perspective of Promotional Communication. *Societies.* 2023;13(1):16. 10.3390/soc13010016

[ref18] Taweekul P : *People’s Participation in Sustainable Tourism Management: A Case Study of Baan Lai Hin, Lai Hin Sub-District, Koh Kha District, Lampang Province.* Graduate School: Chiang Mai University;2001.

