# Supplementary material for: Management of cultural tourism in Ban Toon, Muang District, Phayao Province, Thailand during the COVID-19 pandemic
Source: F1000Res. 2024 Feb 9;13:95. [Version 1] doi: 10.12688/f1000research.134517.1 (PMC11043658; doi:10.12688/f1000research.134517.1)
Supplement: Peer review history

# Reviewer report: Reviewer response for version 1

**DOI:** 10.5256/f1000research.147578.r250188

**Published:** 2024-04-24

**Authors:** Anila Thomas

**Affiliations:** 1 Department of Tourism & Travel Management, Jyoti Nivas College Autonomous, Bangalore, Karnataka, India

**Summary:**

 The research intends to analyze the techniques associated with handling cultural tourism in the community and investigate the economic and social effects on the community, with the assistance of eight major project contributors who play critical roles in the implementation of cultural tourist management. Literary materials give a more complete image of the tourist system, but sources should be contemporary studies and research. The tourism experience variables, which were broadly discussed in the study, must be properly articulated with the most recent data sources.

 This study utilized compiled field notes from interviews and observations, gather data specifications (transcripts), and thematic analysis to acquire the community context in cultural tourism and tourist management components. Participatory Action Research (PAR) was used to fulfil the demands of stakeholders and evaluate tourism routes along these routes. Descriptive statistics were used to examine the development of tourism routes.

 The Ban Toon community in Muang District, Phayao Province, has a strong tourist potential due to its 2010 recognition as a national model village with a self-sufficient economy. The following resources are available:

- The western region offers agricultural learning opportunities.
- The eastern region is a natural and historical tourism destination.
- The ancient port, with cruise operations, is a key community tourist attraction.

At the time of the pandemic, residents in the neighbourhood stopped operating tourism businesses, significantly limiting the chance of visitors visiting Phayao Province and the Red Lotus Sea region.

 The research proposes a new cultural tourism route, and the manner of administering cultural tourism involves community-level groups led by spiritual leaders. The study's findings indicate that the tourists who attempted the route were highly satisfied, leading to the inference that the cultural tourism route "See the mountain, walk through the rice fields, cruise the river" is favoured by both locals who originated the route and tourists who have come to appreciate it.

 Another finding from the study is that incorporating the idea of sustainability in tourism management should take into account the area's management in order to attract visitors while also protecting the area's sustainability. The method to sustainable community tourist management is based on certain components, which include community needs, tourism openness, and tourism management involvement.

 Cultural tourism management in Ban Toon, Muang District, and Phayao Province during the COVID-19 epidemic can provide as a model for other towns with comparable circumstances

Is the work clearly and accurately presented and does it cite the current literature?

Partly

If applicable, is the statistical analysis and its interpretation appropriate?

I cannot comment. A qualified statistician is required.

Are all the source data underlying the results available to ensure full reproducibility?

Yes

Is the study design appropriate and is the work technically sound?

Yes

Are the conclusions drawn adequately supported by the results?

Yes

Are sufficient details of methods and analysis provided to allow replication by others?

Yes

Reviewer Expertise:

Community initiatives and Tourism development, sustainability, SDGs

I confirm that I have read this submission and believe that I have an appropriate level of expertise to confirm that it is of an acceptable scientific standard.

# Reviewer report: Reviewer response for version 1

**DOI:** 10.5256/f1000research.147578.r250190

**Published:** 2024-04-18

**Authors:** I Gusti Bagus Rai Utama

**Affiliations:** 1 Universitas Dhyana Pura, Bali, Indonesia

1. The background of the research is very clear.
2. The research methods used are also appropriate.
3. The research results are not according to the research objectives and methods that have been set. In this section, the author should present the Management of cultural tourism in Ban Toon, Muang District, Phayao Province, Thailand during the COVID-19 pandemic. The results of financial analysis are only as an additional or supporting analysis.
4. In the Conclusion and Discussion section, you should just concentrate on concluding the Management of cultural tourism in Ban Toon, Muang District, Phayao Province, Thailand during the COVID-19 pandemic. For example: how to plan the tour package, what attractions are there and what is attractive to tourists, who is involved in managing it, how to sell it, where is the marketing segmentation, and what is the control system? (POAC: Planning, Organizing, Actuating, and Controlling). Because this research was conducted during the COVID-19 pandemic, how is the health surveillance adaptation system?

Is the work clearly and accurately presented and does it cite the current literature?

Yes

If applicable, is the statistical analysis and its interpretation appropriate?

Yes

Are all the source data underlying the results available to ensure full reproducibility?

Yes

Is the study design appropriate and is the work technically sound?

Yes

Are the conclusions drawn adequately supported by the results?

No

Are sufficient details of methods and analysis provided to allow replication by others?

Yes

Reviewer Expertise:

Economic Tourism, Agritourism, Destination Management, and Senior Tourism

I confirm that I have read this submission and believe that I have an appropriate level of expertise to confirm that it is of an acceptable scientific standard.
